# Towards a Global Interdisciplinary Evidence-Informed Practice: Intimate Partner Violence in the Ethiopian Context

**DOI:** 10.5402/2012/307271

**Published:** 2012-05-20

**Authors:** Sepali Guruge, Amy Bender, Fekadu Aga, Ilene Hyman, Melesse Tamiru, Damen Hailemariam, Andargachew Kassa, Khosro Refaie-Shirpak

**Affiliations:** ^1^School of Nursing, Ryerson University, 350 Victoria Street, Toronto, ON, Canada M5B 2K3; ^2^Faculty of Nursing, University of Toronto, 155 College Street, Toronto, ON, Canada M5T 1P8; ^3^School of Nursing, Addis Ababa University, P.O. Box 9083, Addis Ababa, Ethiopia; ^4^School of Public Health, University of Toronto, 155 College Street, Toronto, ON, Canada M5T 1P8; ^5^School of Public Health, College of Health Science, Addis Ababa University, P.O. Box 9086, Addis Ababa, Ethiopia; ^6^School of Nursing and Midwifery, Hawassa University, P.O. Box 1560, Hawassa, Ethiopia; ^7^Public Health and Preventive Medicine Program, McMaster University, 1280 Main Street West, Hamilton, Ontario, Canada L8S 4K1

## Abstract

*Background*. Intimate partner violence is a global health issue and is associated with a range of health problems for women. Nurses, as the largest health workforce globally, are well positioned to provide care for abused women. *Objectives*. This nursing-led interdisciplinary project was conducted to understand the current state of knowledge about intimate partner violence in Ethiopia and make recommendations for country-specific activities to improve response to intimate partner violence through practice changes, education, and research. *Methods*. The project involved two phases: review of relevant literature and an interdisciplinary stakeholder forum and a meeting with nurse educators. *Findings*. The literature review identified the pervasiveness and complexity of intimate partner violence and its sociocultural determinants in the Ethiopian context. Two significant themes emerged from the forum and the meeting: the value of bringing multiple disciplines together to address the complex issue of intimate partner violence and the need for health care professionals to better understand their roles and responsibilities in actively addressing intimate partner violence. *Conclusions*. Further research on the topic is needed, including studies of prevention and resilience and “best practices” for education and intervention. Interdisciplinary and international research networks can support local efforts to address and prevent intimate partner violence.

## 1. Introduction

 Intimate partner violence (IPV) is defined as the threat of, and/or actual, physical, sexual, psychological, or verbal abuse by a current or former spouse or nonmarital partner [1]. At a global level, IPV occurs in epidemic proportions; the rates of IPV are comparable to those for cancer, cardiovascular diseases, and HIV/AIDS [2]. IPV has been linked to a range of physical and mental health problems [3–7] that may persist long after the violence has ended. Although IPV is considered to be a global public health problem, few health sciences studies have focused on it in low-income countries. This gap is a major impediment to improving the response of local health sectors to the needs of women who are experiencing IPV in these countries and to improving health equity for women everywhere [8]. In particular, the response of nurses as frontline care providers could be improved.

This paper presents the findings from a nurse-led, international, interdisciplinary project aimed at understanding the current situation of IPV in Ethiopia and developing recommendations for country-specific activities to address IPV. In particular, it reports the findings from a review of literature on IPV in Ethiopia, summarizes the process, discussions, and outcomes of an interdisciplinary forum with various stakeholders and a meeting with nursing faculty from several universities across Ethiopia, and makes recommendations for country-specific activities to improve response to IPV in Ethiopia through practice changes, education, and research. The findings may have applications to other low-income countries and/or “marginalized” populations in middle- to high-income countries.

## 2. Background

### 2.1. Ethiopia as Context

Ethiopia is one of the oldest nations in the world and is characterized by great diversity in terms of its topography, climate, ethnicities, and languages, with more than 80 distinct cultural groups. With a population of approximately 75 million and an annual population growth rate of 2.6% (in 2007) [9], Ethiopia is the second most-populous country in Africa. Approximately 84% of its population lives in rural areas, and 52% of the total population is of working age (in 2007) [9]. Since 1975, Ethiopia has experienced wars that have caused approximately 1 in 25 of its citizens to flee to neighbouring countries and elsewhere. It is also considered one of the lowest-income countries in the world, with 65% of the population living below the absolute poverty line. This is reflected in the average life expectancy of 53.42 years for men and 55.42 years for women [9].

The current government is focused on providing access to basic primary health care services to the whole population, using a decentralized system of health centres and health posts. An estimated 75% of urban homes and 42% of rural homes have access to health facilities [10]. A major challenge to providing accessible health services is the inadequate number of sufficiently prepared health care professionals. Based on 2006-07 data, there were 1806 physicians, 792 health officers, 18,146 nurses, 1012 midwives, and 24,571 health extension workers, “giving a health workers ration of 0.27/1000 population" (p.9) [11]. Similarly high patient ratios continue today. As in many other low-income countries, health care professionals face challenges in their work conditions, including inadequate resources. Such broader contextual challenges compound the concern of accurately understanding the roles of various health care professionals and their exact contributions to positive health outcomes for women dealing with IPV, or the health system's interface with other related sectors such as the justice system to address IPV.

## 3. The Project

### 3.1. Objectives

Our collective interest in better understanding IPV in the Ethiopian context evolved from our previous research with Ethiopian immigrants living in Toronto (Hyman and Guruge) [12, 13], an existing Ethiopian-Canadian partnership in graduate nursing education at Addis Ababa University (Aga and Bender), and the interests and work of Hailemariam and Tamiru, public health and health sociology experts, respectively. The objectives of this project were twofold: (1) to understand the current state of knowledge about IPV in Ethiopia and (2) to support country-specific activities to improve responses to IPV through practice changes, education, and research. 

### 3.2. Methods

The project involved two phases. A review of the published literature was first conducted (in Phase  1) to understand the current state of knowledge about IPV in the Ethiopian context. The results of this review served as a launch for subsequent discussions in an interdisciplinary forum and a meeting with nursing faculty held (in Phase 2) in Addis Ababa to develop country-specific activities to improve responses to IPV through practice changes, education, and research. We have outlined the details of each phase below.

#### 3.2.1. Phase 1: A Literature Review

In phase one, a search and review of both academic and grey literature was conducted. The academic search was carried out by project assistants (Kassa and Refaie-Shirpak) with supervision from team members in Canada. MEDLINE and CINAHL databases were searched for literature published from 2000 to 2009, with the following keywords: intimate partner violence, domestic violence, harmful traditional practices, violence against women, abuse, and Ethiopia. The grey literature search, conducted by the Ethiopian project assistant (Kassa) with supervision from Ethiopian team members, included policy documents and statistics produced by or available from university libraries such as Addis Ababa University and governmental and nongovernmental organizations such as the Ethiopian Ministry of Women's Affairs and Ministry of Health. It bears noting that access to such documents was not straight forward, often requiring permissions and presenting timing challenges.

Of the 53 reports collected, 35 were research studies with varying sample sizes (*N* = 396–3,016) using cross-sectional designs, which were conducted in several cities and regions including Meskin and Mareko [14], Addis Ababa [15], Hawassa and Kofale [16], Agaro town [17, 18], Kofele district [19], Arsi Zone Oromia Region [20], and Gondar [21]. Other key documents included a study of seven sub-Saharan African countries involving 15,000 Ethiopian women and 3,092 men [22], and the World Health Organization's (WHO) Multi-country Study on Women's Health and Domestic Violence Against Women [8] and the findings specific to Ethiopia [14].

#### 3.2.2. Phase 2: Addis Ababa Meetings on IPV

Phase 2 was comprised of two meetings held in Addis Ababa in November, 2009: a full-day interdisciplinary forum and a half-day meeting with nursing faculty.

The list of invitees for the interdisciplinary forum was generated by the Ethiopian team members that targeted university departments and specific organizations in health, community, and legal services, as well as appropriate government ministries. Speakers for the forum were also arranged by Ethiopian team members and included researchers/academics and practitioners working in the area of IPV. The invitation letters included a brief description of the project, its aims, a detailed agenda, and logistics of the forum.

The forum was attended by lawyers, nurses, midwives, physicians, academics, and governmental and non-governmental agency staff who are engaged in addressing various aspects of violence against women. At the beginning of the forum, each attendee was given a detailed review of the literature and an executive summary. The following formal presentations were held in the morning: Public Health Perspectives on IPV, Social Services Work in IPV, Legal Services Work in IPV, Women's Advocacy Work in IPV, Socio-cultural Perspectives on IPV and Health, and IPV and HIV/AIDS. These papers were based on research, practice, advocacy, and policy work being undertaken in Ethiopia. Speakers' backgrounds ranged from public health, gender studies, law, psychiatry, and sociology. The afternoon was devoted to small group discussions guided by pre-determined questions on what attendees (*N* = 26) found the least and most surprising in the presentations and in the literature review, and what they thought were common themes as well as important gaps. Each group was asked to develop a list of research priorities, which were reported back to the large group for further discussion.

With three of our six-member project team (The PI (Guruge), the Co-PI (Bender), and a Co-I (Aga)) being nursing faculty, we chose to incorporate a nursing-specific dimension into our project. Because nurses represent the largest group of health care professionals, and nurses may be the only health care professionals immediately available to women in rural contexts, we wanted to learn about nurses' knowledge about IPV and their skills in addressing IPV. Therefore, on the day after the interdisciplinary meeting, we held a meeting with a group of 14 nursing educators from various universities across Ethiopia who were enrolled in the master's program at the Centralized School of Nursing at Addis Ababa University. The group was comprised of eight women and six men who lived and worked in different regions of the country. Eight of the 14 had moved to teaching roles directly after their baccalaureate; only six had both clinical and teaching backgrounds. Their teaching experience ranged from 1 to 20 years, and clinical experience ranged from 0 to 24 years.

## 4. Results

### 4.1. Phase 1: A Literature Review

The literature revealed findings about the forms and prevalence of IPV, health consequences of IPV, risk and protective factors and perceptions of IPV, and responses to IPV.

#### 4.1.1. Forms of IPV

In Ethiopia, IPV is commonly referred to as domestic abuse or domestic violence, wife beating or battering, relationship violence, and spousal abuse. The literature also noted that IPV frequently occurs in the context of marriage or cohabitation, predomestic relationships such as dating relationships, and postdomestic relationships, as in the case of ex-partners who are no longer living together [23]. Various forms of IPV were reported. For example, a national study [24] described wife battering, rape, intimidation, insult and disrespect, denial of food and rest, forced displacement from home, beating and bodily injury, fire burns, forced abortion, abduction, underage marriages, forced labour, and exploitation. Other forms of IPV included slapping, beating, and pushing [19]. Sexual assault and rape by intimate partners were a major concern; one study based on data collected at Adigrat Zonal Hospital from 2000 to 2003 [25] found that of 181 rape victims seeking help at the hospital, approximately 37% identified intimate partners as the perpetrators (ex-husband, 5.6%; boyfriend, 22.1%; ex-boyfriend, 8.3%).

IPV should be understood and addressed within the broader context of violence against women. A number of studies (e.g., [24, 25]) reported that rapes perpetrated by the victim's own relative in a domestic situation accounted for a considerable number of cases.  Table 1 summarizes the different forms of violence against women across women's lifecycle in Ethiopia.

#### 4.1.2. Prevalence of IPV

Several studies reported that IPV in Ethiopia was pervasive. Approximately 90 to 100% of participants in a national study reported that IPV was a common phenomenon in Ethiopia [24]. The lifetime prevalence of any form of IPV ranged from 51 to 78%, with physical and sexual forms being most common. The lifetime and past 12 months prevalence of physical violence ranged from 30 to 60% and 30 to 50%, respectively. The prevalence rates for sexual violence ranged from 35 to 59% over a lifetime and from 30 to 50% during the last 12 months. Only one study examined the prevalence of psychological abuse [21]; it reported a combined prevalence rate of 50.8% for physical, sexual, and/or psychological abuse (i.e., separate rates for psychological abuse were not reported).

Other studies documented the main forms of IPV in different parts of Ethiopia. A study conducted in the Amhara region's Bahir dar/Adet sites [26] found that approximately 64% of participants experienced insults and disrespect, 83% wife battering, 56% intimidation, 72% under age marriage, 64% forced displacement from home, and 55% rape. This study also listed the prevalence of the types of IPV reported to the police: 36% for rape and abduction; 25% for wife beating and bodily injury; 7% for intimidation, insult, and disrespect; 9% for underage marriages; 11% for forced labour and exploitation; 3% for forced displacement from home. A study of gender-based violence and HIV risk in Addis Ababa [27] found that 44% of participants attending antenatal care had experienced physical and/or sexual violence.

In Butajira, the Ethiopian site for WHO's [8] multi-country study (*n* = 2, 261 of ever-partnered women), 48% and 29% of participants reported having experienced physical violence by a current/ex-partner in their lifetime and currently, respectively. Comparatively, rates for sexual violence were 59% and 44.4% (for lifetime and in the last year). The proportion of women reporting either sexual or physical IPV or both was about 71% and 54% (for lifetime and in the last year, resp.). In this regard, Ethiopia has the highest prevalence among the 10 countries included in the WHO study, and for lifetime physical violence alone, Ethiopia ranked second [23].

#### 4.1.3. Health Consequences of IPV

Consistent with the literature from other countries, the literature about IPV in Ethiopia reported a wide range of health consequences from IPV. The main physical health consequences identified were fractures, deep cuts on body parts, injury to eyes and ears [19]; bites and scratches, stabbing, and/or burned body parts; chronic headaches, somatic injuries, dyspepsia and pregnancy complications [21]; sexually transmitted diseases (STDs) [21, 28]; hand fractures, bruises, cuts, loss of body parts or body functions, fistula, trauma to the bladder, urinary incontinence, chronic diseases, vaginal bleeding, and uterine problems [29] and spontaneous abortion and stillbirths, miscarriage, and low-birth-weight babies [30]. A follow-up study of abused women at Adigrat Zonal Hospital (Ammanuel et al. cited in [25]) found that the main presenting symptoms at the first visit were physical injury (30%) and genital injury/problems (41%). At three months after their first visit, 16% presented with pregnancy, 11% abortion, 16% HIV infections, and 14.3% with a positive serum VDRL test (14.3%). At the three-month point, women also reported decreased sexual feeling, aversion to sex, dyspareunia, insomnia, and nightmares among the associated consequences of rape, specifically.

Mental health consequences of IPV were identified [24] including emotional toll [21]; mental distress, suicidal ideation and attempts [18, 25]; depression [14, 18]; stress and anxiety [29]; psychosis [25]. Social consequences of IPV were also identified, such as poverty, prostitution, migration to urban areas, and abduction [18]. These social consequences often extend beyond the victim. For example, studies of street children [31–33] have found that a considerable number of them have witnessed IPV at home; furthermore, being on the street they have experienced rape, unwanted pregnancy, alcohol and substance abuse, and exposure to STDs.

#### 4.1.4. Risk and Protective Factors and Perceptions of IPV

The literature also revealed a number of risk factors for IPV. The risk of physical violence, in particular, was associated with the educational status of women and men, place of residence, religion, arranged marriage, parity, partner's use of alcohol, and the woman's having witnessed family violence as a child [19]. Among the factors contributing to the escalation of violence was substance use/abuse, especially alcohol use/abuse [24].

IPV appears to be tolerated under certain circumstances. For example, Mohamed [34] found that family members justified IPV in the case of girls/young women in an arranged marriage, to ensure that they would be submissive. A study in Oromia and Afar regions (“the case of Fentale and Mille Districts”) found that 52 out of 555 women aged 15–49 years agreed that IPV was justified if a wife refused to have sex with her husband [34]. The *Men and Women in 7 Sub-Saharan African Countries *(including Ethiopia) [22] study reported that an acceptance of wife beating was more prevalent in countries with low levels of female literacy (such as Mali and Ethiopia) compared to those with higher levels. The findings suggested that norms regarding wife beating and gender roles tended to change with socioeconomic development, increasing urbanization, and better education. However, these changes may not be substantial and may be slow to come about.

Intergenerational transmission of patriarchal norms partially explains the gender-based violence that seems to manifest in many forms through the lifecycle [22], so any analysis or intervention of IPV must take into account other forms of violence that take place in a wide range of locations outside the home: schools, neighbourhoods, communities, and societies, where assumptions about femininity and masculinity are enacted.

#### 4.1.5. Responses to IPV

**Women's Responses**

In the WHO [8] study, 61% of abused women in Ethiopia disclosed abuse to informal sources, typically family or friends, and only 45% sought formal help. The most common reasons for not seeking help were violence being seen as “normal,” fear of further violence or losing children, and bringing shame to the family [8]. Gossaye et al. [30] suggested that stigma (particularly that women must have “deserved it”) may contribute to why women rarely report violence to authorities (including health professionals) or talk about it at all. According to a study by the Federal Police of Ethiopia [35], some of the reasons for women's reluctance to report IPV include fear of the partner, further shame, fear of divorce or separation, and lack of knowledge about protective laws. Their report also identified lack of continuous conflict with the partner, feelings of inferiority, and loss of self-worth among abducted women, as well as the stigma of divorce, disability, HIV/AIDS, labour problems and pregnancy as possible factors shaping women's responses to IPV.

Minimal use of formal services may partly reflect a limited availability of services [27]. In addition, the socio-cultural contexts that shape the beliefs often held by Ethiopian health care professionals may impede women's access to care, especially in rural areas where nurses may be the first (or only) health care professional encountered by women experiencing IPV [27, 36]. This raises the question of the health sector's response to IPV.

**The Health Sector's Response**

With the goal of ensuring “health for all,” the current Ethiopian health policy [36] and health sector strategies [37] emphasize decentralization and democratization. Health care delivery in Ethiopia is structured as a tiered system characterized by preventive, promotive, and basic curative services at various levels of administration. While the system strongly focuses on promoting health and preventing illness/injury, IPV is only beginning to be understood and addressed as an important public health issue and receives a limited response in terms of service provision at every tier of the system. According to Gossaye et al. [30], “health providers usually do not consider it as part of their role to screen patients for violence or to provide information or support to women suffering abuse” (p. 38).

Other reasons for limited health service responses to IPV were identified as (1) lack of trained personnel in rural areas, (2) lack of reporting (or a reporting system) of “minor” injuries in the case of rape, (3) delay in the preparation of medical reports (or lacking reports altogether) due to kinship matters or corruption, and (4) expecting the woman to pay for the medical card and write her certificates in reporting the experience to the police [35]. One physician from each of the Polis, Yekatit 12, Arbaminch, and Asella Hospitals were interviewed in one study regarding the problem of limited health care provider response to IPV. The concerns they raised were related to issues such as the time within which a victim should reach a hospital (in order to collect evidence of rape); victims' tendencies to clean themselves following rape; having intercourse with another person (before seeking medical help); delay in seeking help for health consequences of IPV, including unwanted pregnancies; and lack of societal openness to discussing reproductive health issues. A related issue here is that the health and justice systems have overlapping concerns with regard to IPV, notably, in terms of reporting IPV and prosecuting perpetrators of the violence, in keeping with the best interest of the woman who reports. A Federal Police report [35] claimed that the health sector was not responsive to providing police and court systems with adequate evidence to facilitate justice in matters of IPV. The Ministry of Health acknowledged this fact, commenting that the health system has generally fallen short of providing accessible services to women experiencing IPV and of responding to them appropriately and is only beginning to coordinate with law enforcement regarding reporting and referral [9].

#### 4.1.6. Literature Review: A Summary

The literature review highlighted the complexity and insidiousness of IPV—where, how, and to whom it happens, its effects, and the socio-cultural determinants that give root to its pervasiveness. The review also demonstrated that while IPV may receive comparatively little research attention as a health issue in Ethiopia, important studies have been conducted and the findings contribute to the understanding of IPV. More specifically, the review provided our larger project with a solid grounding for the discussions planned in Phase 2 to further critical reflection on the issue itself as well as on the priority needs for moving forward in IPV research and education and enhancing service provision.

### 4.2. Phase 2: Addis Ababa Meetings

#### 4.2.1. Interdisciplinary Forum

The participants provided a critical appraisal of the research conducted to date. While the incidence and prevalence rates of IPV are well documented for Ethiopia, attendees noted that these studies had been carried out in limited areas of the country. Furthermore, while epidemiological data are vital, more qualitative research was perceived as necessary to deepen understanding of women's lived experiences as well as why men commit abusive/violent acts against women. More broadly, public education was seen as critical to bringing about social change in this regard. Several attendees observed that the day focused on negative aspects of IPV and that future work is also needed to build on the successes of existing work being done in terms of awareness raising, violence prevention, and service provision as well as to identify the resilience of the abused women and their children.

Two significant themes emerged from the small group discussions: (1) the value of bringing together clinicians, advocates, educators, and researchers from multiple disciplines to share with and learn from each other and to build consensus on research gaps and identify priorities for interdisciplinary research on IPV in Ethiopia, as IPV spans many disciplines and will not be adequately addressed by individuals working separately within each discipline; and (2) the need for health care professionals to better understand their role and responsibilities in identifying IPV and taking steps to actively address it in practice. These two themes were framed within some assumptions of the need to think globally. Research about IPV in Ethiopia benefits, and is benefitted by, collaborative efforts across countries within Africa and around the world. Suggestions were made about the need to plan more regular forums such as this one and international workshops and seminars addressing IPV.

#### 4.2.2. The Meeting with Nursing Faculty

Discussions in this meeting focused on a set of questions that we developed based on themes from the literature review and issues raised at the interdisciplinary forum, including the following: What is intimate partner violence? How is it understood culturally, locally, in your own town/city? How is it a health issue? How is it an issue for nursing? What should be done about it? What is the nurse's role in caring for women who have experienced IPV? What is the nurse's role in preventing it? What is currently in your nursing school's curriculum on violence, in general, and on IPV, in particular? What should be included in your curriculum?

This was generally a well-informed group of nurses who could easily define IPV and explain its key defining characteristics. Definitions generated by the group fit with those presented earlier in this paper: participants clearly identified the health issues associated with IPV and emphasized education and awareness raising as important strategies in addressing IPV. Like the interdisciplinary group, these nurses also stressed the sociocultural acceptance of the devaluation of women, generally, and IPV, specifically, as contextual elements in the overwhelming rates of violence against women, particularly in more rural areas of the country. As discussion shifted to the education of nursing students in this regard, different interpretations of the countrywide standardized nursing curriculum emerged. While IPV was understood by all to be a four-hour component of the “reproductive health” course, the content of these four hours varied. In the end, participants agreed that Ethiopia's standardized nursing curriculum requires a stronger focus on violence against women to improve assessment, care, and prevention and that the onus is also on nursing schools and individual faculty members to make violence against women more explicit in class content.

#### 4.2.3. Meetings: A Summary

The discussions in both meetings highlighted that, as in most countries around the world, there is a definite need to make the links between health and violence against women more explicit. To delineate this point further, it is vital to raise awareness of IPV amongst health care professionals in Ethiopia, to support them in learning more about IPV and its role in women's health and to generate more coherent strategies for addressing IPV directly with women in care and for guiding them to appropriate resources. More broadly, there is a need to develop a more coordinated systemic response to abused women's needs across sectors, including health and social services, the justice system, housing, economics, and faith communities. Moreover, immediate attention and adequate resources must be directed towards preventing violence and promoting health at community and population levels.

## 5. Discussion

This project identified several gaps in health science research on IPV in Ethiopia, particularly the need for comprehensive interdisciplinary research. At the macrolevel, the diversity of Ethiopia's population and the changes in gender relations brought about by urbanization, globalization, migration, and transient/migratory work require critical analysis of how these affect women's responses to IPV and contribute to their vulnerability and resilience and how these change over time. Specifically, it is important to assess how such changes influence rates and experiences of IPV, and thereby how to best mitigate IPV. Related to this, individual, familial, community, and societal risk and protective factors need to be explored in order to have the greatest effect on the overall incidence of violence.

Important research into the health risks and consequences of IPV has begun; however, more is needed particularly in regions of Ethiopia that have not been captured in previous studies. Since large-scale epidemiological studies mainly capture data on prevalence and risk factors, qualitative research methods can help deepen understandings of, for example, the quality of life of women who are living in or have left abusive intimate relationships. Critical qualitative studies of the perceptions and attitudes of health care professionals toward IPV and the women who seek help can also identify the education, training, and support needs of these professionals.

The need for research in health services is also apparent, specifically, in terms of assessment, treatment interventions, care and support, and prevention. Further study into the assessment of IPV in clinical settings, focussing on the roles and responsibilities of health care providers other than physicians, can be a first step in the development of standardized screening tools for health clinics and hospitals. A more complete understanding of the accessibility issues faced by Ethiopian women seeking IPV-related services is needed to improve intersectoral collaboration at the point of contact. Finally, the role and the types of public education that can effectively challenge gender norms need to be examined in the interest of developing effective violence prevention campaigns.

In addition, our project identified the importance of focusing on practice and the education of health care professionals. Health care practitioners, from physicians and nurses to health extension workers and volunteers, need adequate and appropriate education about IPV. This includes learning how best to care for women in open, non-judgmental, and supportive ways and developing skills in therapeutic communication regarding the topic of IPV and advocacy.

Lastly, the findings of this project highlight the critical importance of (1) an interdisciplinary approach to this work, (2) systematic access to information as a starting point, and (3) being careful to reflect on assumptions of what is “known” about a topic or issue, particularly from an international perspective. This project brought people together who had never met before and created a starting point in working towards improving local responses to IPV through practice changes, education, and research.

## 6. Conclusion

Interdisciplinary research and practice initiatives can have far-reaching effects in understanding and addressing IPV. Nursing, medicine, public health, and law, as well as the social sciences, political science, gender studies, and the humanities all have dovetailing research interests in IPV that can lead to effective prevention and intervention programs. By adding international collaboration to this mix, through the creation of partnerships across countries, universities, and health care settings, IPV may be better understood as a global health issue and thereby the health of women in Ethiopia and the diaspora improved.

## Figures and Tables

**Table 1:** Different forms of violence occurring in Ethiopian women's lifecycle [19, 22, 24, 25, 31, 34].

Phase	Type of violence
Prebirth	Male preference
Infancy	Preferential treatment for boys (e.g., stopping breastfeeding earlier for girls), female genital mutilation (FGM)
Childhood	Food/nutritional misallocation, FGM, early marriage, sexual abuse, rape
Adolescence	Food/nutritional misallocation, FGM, not sending girls to school, psychological abuse, sexual harassment, domestic violence (IPV), abduction, rape, trafficking
Adulthood	Food/nutritional misallocation, domestic violence (IPV), sexual harassment, rape
Old age	Food/nutritional misallocation, rape

## References

[1] United Nations Declaration on the elimination of violence against women.

[2] Heise LL, Pitanguy J, Germain A Violence against women: the hidden health burden.

[3] Eby KK, Campbell J, Sullivan C, Davidson W (1995). Health effects of experiences of sexual violence for women with abusive partners.. *Health Care for Women International*.

[4] Campbell JC, Lewandowski LA (1997). Mental and physical health effects of intimate partner violence on women and children. *Psychiatric Clinics of North America*.

[5] Coker AL, Smith PH, Bethea L, King MR, McKeown RE (2000). Physical health consequences of physical and psychological intimate partner violence. *Archives of Family Medicine*.

[6] Velzeboer M, Ellsberg M, Arcas CC, Garcia-Morano C (2003). *Violence against Women: The Health Sector Responds*.

[7] Humphreys J, Sharps PW, Campbell JC (2005). What we know and what we still need to learn. *Journal of Interpersonal Violence*.

[8] World Health Organization (2006). Multi-country study on women’s health and domestic violence against women. *Summary report: initial results on prevalence, health outcomes and women’s responses*.

[9] Federal Democratic Republic of Ethiopia Ministry of Health (2006). National reproductive health strategy, 2006–2015: the social and institutional parameters of women’s health.

[10] World Bank Group http://web.worldbank.org/wbsite/external/countries/africaext/ethiopiaextn/0,menuPK:295935~pagePK:141159~piPK:141110~theSitePK:295930,00.html.

[11] World Health Organization (WHO) WHO country cooperation strategy 2008–2011 Ethiopia. http://www.who.int/countryfocus/cooperation_strategy/ccs_eth_en.pdf.

[12] Hyman I, Guruge S, Mason R (2004). Post-migration changes in gender relations among ethiopian couples living in Canada. *Canadian Journal of Nursing Research*.

[13] Hyman I, Guruge S, Mason R (2008). The impact of post-migration changes on marital relationships: a study of Ethiopian immigrant couples in Toronto. *Journal of Comparative Family Studies*.

[14] Deyessa N, Berhane Y, Alem A (2009). Intimate partner violence and depression among women in rural Ethiopia: a cross-sectional study. *Clinical Practice and Epidemiology in Mental Health*.

[15] Amdie G (2005). *Gender based violence and risk of HIV infection among women attending VCT service in Addis Ababa City*.

[16] Eyoel M (2007). *Patterns of intimate partner violence among married women of Awassa Town*.

[17] Hundie A (2007). *Cause and consequences of early marriage among Selale Oromo women: the case of Hindabu Abote and Kuyu Woredas, North Shewa Zone, Oromia National Regional State*.

[18] Tadegge AD (2008). The mental health consequences of intimate partner violence against women in AgaroTown, southwest Ethiopia. *Tropical Doctor*.

[19] Dibaba Y (2008). Prevalence and correlates of intimate partner physical violence against women in Kofale District, Ethiopia. *Tropical Doctor*.

[20] Kedir H, Deyessa N, Berhane Y Magnitude and immediate outcome of partner physical violence.

[21] Yigzaw T, Yibrie A, Kebede Y (2004). Domestic violence around Gondar in north-west Ethiopia. *Ethiopian Journal of Health Development*.

[22] ManjuRani BS, Bonu S, Diop-Sidibé N (2004). An empirical investigation of attitudes towards wife-beating among men and women in seven sub-Saharan African countries.. *African Journal of Reproductive Health*.

[23] Gizaw B (2002). *Some Reflection on Criminalizing Domestic Violence Against Women With Emphasis in Ethiopia*.

[24] Ethiopian Women Lawyers Association (2008). *Nationwide Survey on Domestic Violence*.

[25] Gessessew A, Mesfin M (2004). Rape and health problems in Adigrat Zonal Hospital. *Ethiopian Journal of Health Development*.

[26] Muleta M, Williams G (1999). Postcoital injuries treated at the Addis Ababa Fistula Hospital, 1991–97. *Lancet*.

[27] Demissie W (2007). *Gender based violence and risk of HIV infection among women attending ANC service HIV sentinel surveillance sites in Addis Ababa, Ethiopia*.

[28] Tegegne H (2007). *The interface between violence against women and HIV/AIDS: the experience of HIV positive women beneficiaries of the Society for Women and AIDS in Africa-Ethiopia*.

[29] Mizana S, Ararsa F, Tadesse B, Workineh B YesetLig Telefa be Kofele ena DoDola Woredawoch: Nehase 1996 Adama.

[30] Gossaye Y, Deyessa N, Berhane Y, Ellsberg M, Emmelin M (2003). Butajira rural health program: women’s life events study in rural Ethiopia. *Ethiopian Journal of Health Development*.

[31] Lalor KJ (1999). Street children: a comparative perspective. *Child Abuse & Neglect*.

[32] Pathfinder International Ethiopia Gender mainstreaming in reproductive health, family planning and HIV/AIDS programs.

[33] Wondimu H, Terefe H, Abdi YO *Gender and Cross Cultural Dynamics in Ethiopia: A Study of Eleven Ethnic Groups*.

[34] Mohamed A (2007). *Violence against women and girls in the pastoralist communities of Oromia and Afar regions: the case of Fentale and Mille Districts*.

[35] Federal Police of Ethiopia Research and Investigation Guidance Office Yeasgededo Medferena, Telefa, Wonjel, Menseawochachew ena Yemiasketeluwachew Gudatoch Beethiopia.

[36] Transitional Government of Ethiopia (1993). *National Policy on Ethiopian Women*.

[37] Transitional Government of Ethiopia (1995). *Health Sector Strategy*.

